# The Relationship Between Childhood Neglect and Malevolent Creativity: The Mediating Effect of the Dark Triad Personality

**DOI:** 10.3389/fpsyg.2020.613695

**Published:** 2020-12-16

**Authors:** Xuji Jia, Qingjin Wang, Lin Lin

**Affiliations:** ^1^Key Research Base of Humanities and Social Sciences of the Ministry of Education, Academy of Psychology and Behavior, Tianjin Normal University, Tianjin, China; ^2^Faculty of Psychology, Tianjin Normal University, Tianjin, China; ^3^Tianjin Social Science Laboratory of Students’ Mental Development and Learning, Tianjin, China

**Keywords:** malevolent creativity, childhood neglect, the dark triad, mediating effect, creativity

## Abstract

In addition to what we know as benevolent creativity, which involves originality and usefulness, creativity also includes malevolent creativity, which involves the application of creative ideas to intentionally harm others. This study aimed to explore the environmental and individual predictors of malevolent creativity. We investigated the relationship among childhood neglect, Dark Triad personality traits and malevolent creativity and examined the mediating role of Dark Triad personality. A large sample (*N* = 991) of Chinese undergraduate students completed the childhood neglect scale, the 12-item Dirty Dozen and the Malevolent Creativity Behavior Scale. Structural equation modeling demonstrated that childhood neglect was positively related to individual malevolent creativity, and the Dark Triad partially mediated this relationship. Additionally, gender differences were found, such that childhood neglect had a stronger effect on malevolent creativity through the Dark Triad among males than females. The results were discussed from the perspectives of life history theory and social information processing theory.

## Introduction

Creativity has valuable and beneficial effects on social development and the quality of personal life. The traditional definition of creativity focuses on the originality and usefulness of people’s creative products, which represent benevolent creativity (Gong et al., 2016). However, creativity also has a dark side. Cropley et al. (2010) published a related monograph The Dark Side of Creativity, which gained widespread attention. Rogers (1959) pointed out that the dark side of creativity could have both positive and negative purposes; thus, malevolent creativity and negative creativity can be distinguished. Malevolent creativity is defined as creativity that is deliberately planned to damage others (Cropley et al., 2014). The relevant and practical importance of malevolent creativity has been validated and further developed in the area of terrorism and crime (Cropley and Cropley, 2011; Gill et al., 2013). The research on malevolent creativity not only contributes to people’s comprehensive understanding of creativity but, more importantly, warns people that creativity driven by malicious purposes may cause great harm to individuals and society. Therefore, academic research on malevolent creativity has great social value.

Previous studies have shown that both environmental and individual factors have an impact on the development of malevolent creativity (James et al., 1999; Gong and Liu, 2016). On the one hand, James et al. (1999) pointed out that social climate, cultural atmosphere, and social complexity are related to malevolent creativity. For instance, unfair social situations provoked more malevolent solutions for problem-solving tasks (Clark and James, 1999), and threatening social circumstances evoked malevolent creative responses for divergent thinking tests (Baas et al., 2019). On the other hand, a review showed that personality and emotion also have close relationships with the generation of malevolent creativity (Gong and Liu, 2016). For example, participants with high levels of aggression and low levels of conscientiousness exhibited more malevolent creativity (Lee and Dow, 2011), and emotional intelligence negatively predicted participants’ expression of malevolent creativity (Harris et al., 2013). Additionally, all of the above studies also showed that everyone might have the potential to demonstrate malevolent creativity, as it is not exclusive to criminals and terrorists. In the present study, we investigated the environmental and individual predictors of malevolent creativity in the general population with the aim of controling, intervening and reducing the expression of malevolence in the long run.

“Childhood neglect” has been defined as the “neglectful” failure of caregivers to meet the needs of a child without motive while being unaware of the harm being caused (Golden et al., 2003). Neglect is one of the four internationally recognized types of child maltreatments (the others are physical abuse, sexual abuse, and emotional abuse), and based on the limited findings available, the consequences of child neglect are as serious as those of all other types of maltreatments and witnessing domestic violence (Trickett and McBride-Chang, 1995; Hart et al., 1998). Childhood neglect is likely to fundamentally impact individuals’ cognitive, social-emotional, and behavioral development (Hildyard and Wolfe, 2002). These negative effects often endure through adolescence and adulthood (Odgers et al., 2008).

Childhood neglect may exert an effect on malevolent creativity in terms of cognitive and emotional aspects. For example, neglected children have been shown to be the most unhappy group of children (Hildyard and Wolfe, 2002), and childhood neglect generally increases stress sensitivity (Harkness et al., 2006), which predicts depressive symptoms within adults (Infurna et al., 2016). When feeling negative, individuals become more inward-focused, more analytical, and process information in a more bottom-up fashion, engendering cognitive persistence (De Dreu et al., 2012). Individuals with highly detail-oriented analysis may better recognize deviance opportunities and an inward-focused and persistent thinking style may encourage individuals to construct more cautious and successful strategies to capitalize on these opportunities (Grubb and McDaniel, 2007; Gamman and Raein, 2010) and thus may promote malevolent creativity. In addition to emotional valence, emotional intelligence has been found to be negatively correlated with malevolent creativity measured by both the problem-solving task and the divergent thinking paradigm (Harris et al., 2013). Specifically, children with histories of neglect generally have deficits in identifying emotions and reflecting on emotional experiences (Edwards et al., 2005), and these deficits in emotion processing and regulation persist into adulthood (Young and Widom, 2015; Jennissen et al., 2016), which may influence the development of malevolent creativity.

Moreover, childhood neglect may influence the development of malevolent creativity from social aspects. Generally, safe and optimal family environments, such as having a high socioeconomic status and having involved parents with warmth and structure parenting styles, have been proven to contribute to the development of benevolent creativity (Dai et al., 2012; Jankowska and Karwowski, 2018; Moltafet et al., 2018). However, detrimental childhood experiences, such as poor parental care or high parent-child conflict, affect personality development and create a more distrustful, malicious interpersonal style (Csathó and Birkás, 2018). For instance, childhood exposure to family neglect was positively associated with exploitation and retaliatory defection of an interaction partner (McCullough et al., 2013). Similarly, a longitudinal study showed that chronic childhood neglect predicted later aggression or delinquency bolstering that neglect impairs social functioning broadly (Logan-Greene and Semanchin Jones, 2015). Thus, childhood neglect is a risk factor for adolescents, which may reduce prosocial behavior (Llorca et al., 2017) and predispose individuals to think, believe, and perceive in a malevolently biased way (Anderson and Bushman, 2002).

The Dark Triad is consisted of Machiavellianism, psychopathy and narcissism, which are three personality traits interconnected but conceptually independent of each other (Paulhus and Williams, 2002). Individuals with high levels of Machiavellianism are lack of empathy and good at strategy and manipulation (Jonason et al., 2013a; Akram et al., 2018); psychopathy is characterized by impulsivity, lack of control, interpersonal antagonism and deficits in affect (Palmer et al., 2017; Akram et al., 2018); narcissism involves a sense of excellence, self-absorbed, and entitlement (Jonason et al., 2013b; Sabouri et al., 2016). The general view is that the Dark Triad personality traits represent the malevolent side of human nature and thus are inherently maladaptive and accompanied by negative psychosocial consequences (e.g., aggression, delinquency, and cyberbullying; Muris et al., 2017; Moor and Anderson, 2019).

Generally, in the field of the association between personality and creativity, most researchers examined benevolent creativity from the socially desirable aspect of personality (e.g., the Big Five traits; McCrae, 1987; Feist, 1993, 1998; Gelade, 2002), while few studies revealed the dark side of creativity from the perspective of personality. We infer that the Dark Triad might be associated with malevolent creativity based on limited studies. First, the Dark Triad (Jonason et al., 2013c) and malevolent creativity (Lee and Dow, 2011) are separately connected with different dimensions of the Big Five personality, which may signify some shared variance among them. Second, some evidences suggest that Machiavellianism, psychopathy and malevolent creativity are positively connected (Jonason et al., 2017), and that the Dark Triad might be bound up with forms of creation (Kapoor, 2015). Third, individuals with high levels of malevolent creativity may be better at telling more convincing lies (Hao et al., 2016), acting more creatively and criminally (Cropley et al., 2008; Eisenman, 2008), and showing lower emotional intelligence (Harris et al., 2013), all of which are closely related to the Dark Triad (Jonason and Webster, 2012; Jonason and Krause, 2013; Baughman et al., 2014). Taken together, evidences indicate a possible and plausible link between malevolent creativity and the Dark Triad.

Although the Dark Triad constructs share the core elements of callousness and hostile, they are distinct from each other (Paulhus and Williams, 2002). Therefore, they may have different effects on malevolent creativity. For example, Machiavellianism and psychopathy appear to be the “darker” shades of the Triad (Jonason et al., 2015b) because the aggressive, deceptive, and antisocial nature may result in a destructively biased form of creative expression (Jonason et al., 2012a; Baughman et al., 2014). Theoretically, Machiavellian individuals are thought to be strategic manipulators and callous pragmatists demonstrating behavioral flexibility (Hawley, 2006) and average or above-average impulse control (Miller et al., 2016). At the same time, original thinkers can be more morally flexible and dishonest than others (Gino and Ariely, 2012), and highly malicious creative people show better capability of impulse control than lowly ones (Gong et al., 2017). Therefore, Machiavellianism and malevolent creativity may share some common features. However, psychopathy was associated with dysfunctional impulsivity, whereas narcissism was correlated with functional impulsivity (Jones and Paulhus, 2011), which means psychopathy involves poor self-regulation and different cognitive deficits that may undermine creative outputs (Jonason et al., 2015a), but narcissism involves venturesome social engagement, which is required to generate novel ideas. Thus, we assume that Machiavellianism, psychopathy and narcissism may exert their effects on creativity to varying degrees.

Life history theory proposed by Kaplan and Gangestad (2005) predicts that the Dark Triad personality traits may cluster in a non-random fashion in response to the unpredictable and harsh conditions related to social-ecology in childhood (Jonason et al., 2014; Csathó and Birkás, 2018). First, high levels of unpredictable and harsh environments in childhood coupled with the scarcity of resources favor faster life history strategies for accelerating physiological development and an emphasis on immediate gains (Ellis, 2004; Figueredo et al., 2006; Belsky et al., 2010). Then, behavioral indicators of fast life history strategy may emerge, such as opportunistic or exploitative action, inimical attitude, and poor social skills (de Baca et al., 2016; Chang and Lu, 2018), which are common features of Machiavellianism, psychopathy, and narcissism. Finally, to some extent, the Dark Triad traits could be regarded as a synthesis of personality index for fast life history strategies (Jonason et al., 2012b; McDonald et al., 2012), which means personality directs cognitive-affective reactions, socioemotional responses, and behavioral adaptations to current contexts. Taken together, we hypothesize that the Dark Triad may also be related to childhood neglect.

Moreover, interactionist model of creativity (Woodman and Schoenfeldt, 1990) afford us a framework for understanding individual differences in creative behavior, which incorporate antecedent conditions (e.g., early socialization, family position), person variables (e.g., cognitive style, personality) and situation variables. In terms of the interactionist model, antecedent conditions affect the development of an individual’s personality and cognitive style and then contribute to define individual’s existing situation at any given time, which may make individuals produce creative behavior. In the present study, individuals who suffered childhood neglect tend to experience negative emotions (Hildyard and Wolfe, 2002; Infurna et al., 2016), low emotional intelligence and deficits in recognition and regulation emotions (Edwards et al., 2005; Jennissen et al., 2016), thus developing a distrustful, malicious interpersonal style (Csathó and Birkás, 2018) and creating self-centered, callous, and manipulative personality traits (de Baca et al., 2016; Chang and Lu, 2018). According to the interactionist model, detrimental childhood family conditions interact with malicious personality to contribute to define the existing situation and predispose individual to think and react in a malevolence way. Another perspective to understand the present study is that individuals who have experienced childhood neglect display inward-focused and persistent cognitive styles due to negative emotional states (De Dreu et al., 2012), and better recognize and capitalize on deviance opportunities due to detail-oriented processing and cautious strategy construction (Grubb and McDaniel, 2007; Gamman and Raein, 2010). Individuals with dark personality traits, who suffered childhood neglect, may benefit from the cognitive style of persistence and flexibility described above, develop creativity higher than average people (Nijstad et al., 2010), and generate more useful and original ideas to harm others.

In addition, evidence suggests that there are significant gender differences in both the Dark Triad personality traits (Muris et al., 2017) and malevolent creativity (Lee and Dow, 2011; Harris and Reiter-Palmon, 2015; Dumas and Strickland, 2018). Generally, at a young age, boys often exhibit more conduct problems, delinquency, and violence than girls do (Cale and Lilienfeld, 2002), and the gender difference continues into adulthood (Cale and Lilienfeld, 2002). Furthermore, a meta-analysis and critical review of the literature showed that Dark Triad traits are more prevalent among men than women (Muris et al., 2017). Similarly, Lee and Dow (2011) found that male participants generated significantly more malevolent responses to the alternate uses task than women did, and the effect was replicated and extended to other malevolent divergent thinking tasks (Harris and Reiter-Palmon, 2015; Dumas and Strickland, 2018). Thus, we assume in the present study that male participants who perceived childhood neglect were more likely to develop Dark Triad personality traits and engage in malevolent creativity behavior than females.

In summary, numerous studies have indicated that superior or inferior family factors promote or hinder the development of general creativity in individuals (Jankowska and Karwowski, 2018; Moltafet et al., 2018). It is obvious that the family environment plays an important role in the development of creativity, but no research has yet explored the influence of the family environment or childhood experience on malevolent creativity. Furthermore, the Dark Triad personality traits have a close relationship with childhood adversity and the dark side of creativity. Therefore, the purpose of the present study was to investigate the effect of childhood neglect on malevolent creativity and the mediating role of the Dark Triad personality traits in the relationship between them. Additionally, there may be gender differences in the mediating effect. Thus, the following hypotheses were proposed:

Hypothesis 1. Childhood neglect would be positively associated with malevolent creativity.
Hypothesis 2. The Dark Triad personality traits mediate the relationship between childhood neglect and malevolent creativity.
Hypothesis 3. The relationship among childhood neglect, Dark Triad traits and malevolent creativity would be significantly stronger for male participants than for female participants.

**FIGURE 1:**
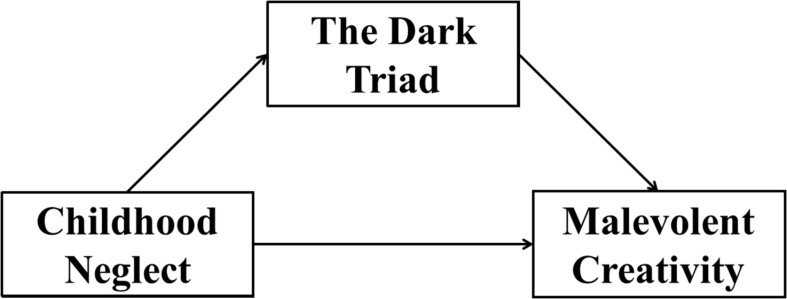
Hypothesized model.

The proposed integrated model is illustrated in Figure 1.

## Materials and Methods

### Participants

The participants were Chinese undergraduate students mainly from Hebei and Sichuan provinces. After excluding participants with invalid data, 991 respondents remained, including 236 males (23.8%) and 755 females (76.2%). A total of 390 respondents (39.4%) were 1st-year students, 537 (54.2%) were 2nd-year students, 11 (1.1%) were 3rd-year students, 37 (3.7%) were 4th-year students, and 16 (1.6%) were 5th-year students (medicine and architecture are 5-year majors). A total of 175 (17.7%) were majoring in science, 496 (50.1%) were majoring in literature, 189 (19.1%) were majoring in engineering, and 131 (13.2%) were majoring in art.

### Measures

#### Childhood Neglect

Childhood neglect was assessed with the Child Psychology Abuse and Neglect Scale (CPANS; Pan et al., 2010), which has been employed in Chinese samples and shows good reliability and validity (Wu et al., 2011; Song and Liu, 2013). Childhood neglect is one of the CPANS subscales. The instrument consists of 17 items, with nine items assessing emotional neglect (e.g., “my parents don’t comfort me when I’m sad or afraid”), four items assessing educational neglect (e.g., “my parents don’t take me to interesting places where I can increase my knowledge”), and four items assessing physical/supervisory neglect (e.g., “when I go out, my parents don’t care about where I go or who I hang out with”). Participants rated the items from one (never) to five (always). Higher scores are signified by higher levels of childhood neglect. In the present sample, Cronbach’s alphas were 0.75 for emotional neglect, 0.73 for educational neglect, 0.63 for physical/supervisory neglect, and 0.87 for the entire scale.

#### The Dark Triad

12-item Dirty Dozen (Jonason and Webster, 2010) which have translated into Chinese version by Geng et al. (2015) were adopted to assess the level of Dark Triad. The scale includes three subscales and four items for each subscale: Machiavellianism (e.g., “I tend to manipulate others to get my way”), psychopathy (e.g., “I tend to lack remorse”), and narcissism (e.g., “I tend to seek prestige or status”). Scores were averaged to create three subscale scores, and higher scores indicated higher levels of the subscale personality trait. This scale has been used to assess the level of Dark Triad traits for Chinese groups (Geng et al., 2017, 2018). In the present sample, the Cronbach’s alphas were 0.80 for Machiavellianism, 0.60 for psychopathy, and 0.75 for narcissism.

#### Malevolent Creativity

We assessed malevolent creativity using the Malevolent Creativity Behavior Scale (MCBS), developed by Hao et al. (2016). The scale has 13 items and three subscales with six items assessing hurting people (e.g., “How often do you think about ideas to take revenge when being unfairly treated”), four items assessing lying (e.g., “How often do you fabricate lies to simplify a problem situation”), and three items assessing playing tricks (e.g., “How often do you have ideas about how to pull pranks on others”). The response options varied from one (never) to five (always). This scale has been illustrated good reliability and validity in different samples of Chinese (Fang, 2017; Wang, 2018). In the present sample, Cronbach’s alphas were 0.77 for hurting people, 0.84 for lying, 0.75 for playing tricks, and 0.89 for the entire scale.

### Procedure

This project was approved by the Research Ethics Committee of Tianjin Normal University and complied with the Declaration of Helsinki involving human subject. An online questionnaire was adopted to assess the level of childhood neglect, the Dark Triad personality and malevolent creativity. Prior to testing, participants were given an online link containing the online informed consent. After they confirmed informed consent, the online survey would go on. If participants declined to participate, the survey ended. The rights as study participants were fully informed in the form of electronic text. We informed participants that completing the surveys was completely voluntary, that they had a right to refuse to complete the surveys or drop out of the research at any time, and that the results would remain confidential.

### Statistical Analyses

First, we summarized the correlations among childhood neglect, Dark Triad personality traits and malevolent creativity using SPSS 20 software. Then, we performed structural equation modelling (SEM) to investigate the impact of Dark Triad personality traits on the relationship between childhood neglect and malevolent creativity using Mplus 7.0 software (Muthén and Muthén, 1998). The robust maximum likelihood (MLR) estimator was used to account for the identified non-normality of the data. The following indices were used to examine the model’s data fit: the Tucker-Lewis index (TLI), the comparative fit index (CFI), the root mean square error approximation (RMSEA), and the standardized root mean square residual (SRMR). In addition, TLI, CFI > 0.90, and RMSEA, SRMR < 0.08 indicated the model fitted well (Hu and Bentler, 1999). After the final model was determined, bias-corrected bootstrapping was adopted to verify the significance of the mediating effects, which has provided with greater statistical power than traditional analysis of mediation (MacKinnon et al., 2004). None of the 95% confidence intervals including zero means a significant mediating. In our study, 1,000 bootstrap samples were randomly sampled and replaced from the dataset.

## Results

### Descriptive Statistics and Correlations Among the Variables

The means, standard deviations and correlation coefficients among the study variables are displayed in Table 1. Pearson’s correlations showed that childhood neglect, Dark Triad personality traits and malevolent creativity were all significantly positively correlated with each other (*p* < 0.001). In addition, the Dark Triad personality traits and malevolent creativity were negatively associated with gender separately (*p* < 0.001), indicating a higher level of dark traits and malevolent creativity for males, while there was no significant association between childhood neglect and gender (*p* > 0.05).

**TABLE 1:** Mean, Standard deviations, and correlations among study variables (*N* = 991).

**Variables**	**1**	**2**	**3**	**4**	**5**	**6**	**7**	**8**	**9**	**10**	**11**	**12**
(1) Childhood neglect	1											
(2) Emotional neglect	0.89***	1										
(3) Educational neglect	0.91***	0.73***	1									
(4) Physical/supervisory neglect	0.88***	0.67***	0.67***	1								
(5) Machiavellianism	0.25***	0.25***	0.21***	0.20***	1							
(6) Psychopathy	0.33***	0.32***	0.29***	0.26***	0.65***	1						
(7) Narcissism	0.18***	0.20***	0.18***	0.11**	0.47***	0.44***	1					
(8) Malevolent creativity	0.26***	0.28***	0.23***	0.19***	0.63***	0.50***	0.48***	1				
(9) Hurting people	0.22***	0.23***	0.20***	0.15***	0.58***	0.45***	0.38***	0.82***	1			
(10) Lying	0.24***	0.25***	0.22***	0.18***	0.57***	0.43***	0.47***	0.89***	0.60***	1		
(11) Playing tricks	0.22***	0.23***	0.19***	0.17***	0.51***	0.42***	0.39***	0.88***	0.62***	0.66***	1	
(12) Gender^a^	0.02	−0.01	0.45	0.07	−0.24***	−0.15***	−0.13***	−0.25***	−0.23***	−0.20***	−0.22***	1
*M*	1.98	2.04	1.96	1.92	1.44	1.70	2.89	1.68	1.55	1.84	1.64	1.76
*SD*	0.65	0.65	0.80	0.74	0.59	0.64	0.86	0.56	0.52	0.73	0.67	0.43

^*a*^Male = 1, female = 2. *p < 0.05, **p < 0.01, ***p < 0.001.

### Measurement Model

We first tested the data fit of the measurement model using confirmatory factor analysis. The measurement model included three latent variables (childhood neglect, Dark Triad personality traits, and malevolent creativity) and nine observed variables. All the indices of the measurement model showed a good data fit: χ^2^ = 94.96 (*p* < 0.001), df = 24, χ^2^/df = 3.96, CFI = 0.98, TLI = 0.97, RMSEA = 0.06 [90% CI = (0.04, 0.07)], SRMR = 0.03, and all factor loadings for the indicators of the latent variables were significant (*p* < 0.001). The results showed that all latent factors were well represented by their respective indicators.

### Structural Model

A structural equation model was adopted to examine the mediating roles of Dark Triad personality traits in the relationship between childhood neglect and malevolent creativity. Furthermore, because females were predominant in the current study and the correlations between gender and Dark Triad personality traits and malevolent creativity were significant, we incorporated gender as a control variable. The results showed that the fit indices indicated a good model fit:χ^2^ = 56.67 (*p* < 0.05), df = 25, χ^2^/df = 2.27, CFI = 0.99, TLI = 0.98, RMSEA = 0.04 [90% CI = (0.02, 0.05)], SRMR = 0.02. Additionally, a reverse model was tested to assess whether the Dark Triad had an effect on malevolent creativity through perceived childhood neglect and found that childhood neglect only significantly mediated the relationship between psychopathy and malevolent creativity. Furthermore, since the Dark Triad mediating model has greater theoretical and empirical support than the reverse model (Jonason et al., 2014; Csathó and Birkás, 2018; Liu et al., 2019), the hypothesized model was regarded as the preferable of the two in describing relationships among the variables.

**FIGURE 2:**
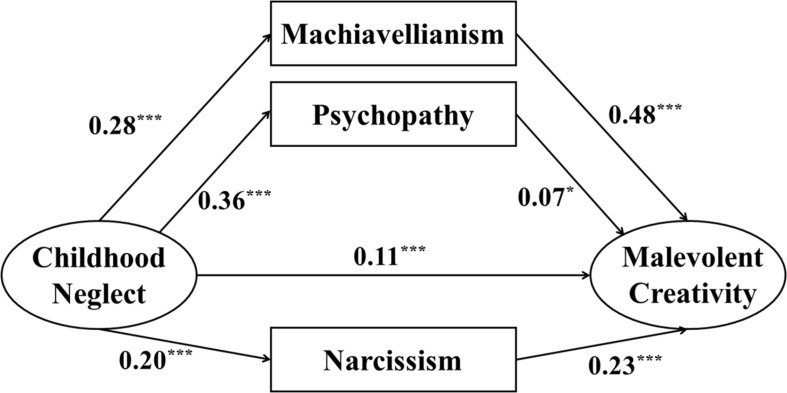
The SEM analysis conducted to test the pathways among childhood neglect, Dark Triad personality traits and malevolent creativity (*N* = 991). All paths are standardized, and the control variable are not included in the presentation of the model. *p < 0.05, ***p < 0.001.

Childhood neglect significantly positively predicted Machiavellianism (β = 0.28, *p* < 0.001), psychopathy (β = 0.36, *p* < 0.001), and narcissism (β = 0.20, *p* < 0.001). Machiavellianism (β = 0.48, *p* < 0.001), psychopathy (β = 0.07, *p* < 0.05), and narcissism (β = 0.23, *p* < 0.001) significantly positively predicted malevolent creativity. The direct effect of childhood neglect on malevolent creativity was significant (β = 0.11, *p* < 0.001). Therefore, the results of the indirect effects demonstrated that Machiavellianism (β = 0.13, *p* < 0.001), psychopathy (β = 0.03, *p* < 0.05), and narcissism (β = 0.05, *p* < 0.001) mediated the relationship between childhood neglect and malevolent creativity. Furthermore, bootstrapping tests indicated that the mediating effects were significant for Machiavellianism [95% CI = (0.100, 0.171)], psychopathy [95% CI = (0.002, 0.057)], and narcissism [95% CI = (0.029, 0.067)] (see Figure 2 and Table 2).

**TABLE 2:** Standardized direct and indirect pathway of the model (*N* = 991).

**Model pathways**	**ß**	***P***	**95% CI**
**Direct effect**			
Childhood neglect→MC	0.11	<0.001	[0.043, 0.167]
**Indirect effect**			
Childhood neglect→	0.13	<0.001	[0.100, 0.171]
Machiavellianism→MC			
Childhood neglect→psychopathy→MC	0.03	<0.05	[0.002, 0.057]
Childhood neglect→narcissism→MC	0.05	<0.001	[0.029, 0.067]
Total indirect effect	0.21	<0.001	[0.160, 0.255]

*MC, malevolent creativity; CI, confidence interval.*

**FIGURE 3:**
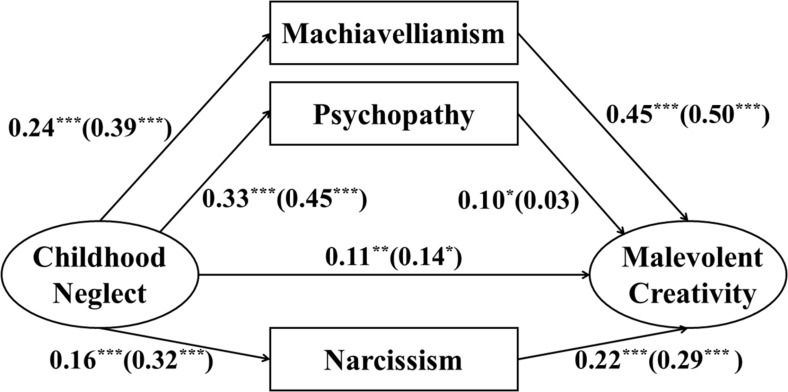
The SEM analysis conducted to test the pathways among female (male) participants. All paths are standardized. *p < 0.05, **p < 0.01, ***p < 0.00.

Then, we examined the SEM for males and females. For females, childhood neglect significantly positively predicted Machiavellianism (β = 0.24, *p* < 0.001), psychopathy (β = 0.33, *p* < 0.001), and narcissism (β = 0.16, *p* < 0.001). Machiavellianism (β = 0.45, *p* < 0.001), psychopathy (β = 0.10, *p* < 0.05), and narcissism (β = 0.22, *p* < 0.001) significantly positively predicted malevolent creativity. The direct effect of childhood neglect on malevolent creativity was significant (β = 0.11, *p* < 0.01). Therefore, the results of the indirect effects demonstrated that Machiavellianism (β = 0.12, *p* < 0.001), psychopathy (β = 0.03, *p* < 0.05), and narcissism (β = 0.04, *p* < 0.01) partially mediated the relationship between childhood neglect and malevolent creativity. Bootstrapping tests indicated that the mediating effects of Machiavellianism, psychopathy, and narcissism among females were significant [95% CI = (0.071, 0.150) for Machiavellianism, 95% CI = (0.004, 0.067) for psychopathy and 95% CI = (0.019, 0.061) for narcissism; see Figure 3 and Table 3].

**TABLE 3:** Standardized direct and indirect pathway of the model among female and male participants.

**Model pathways**	**ß**	***P***	**95% CI**
**Females (*N* = 755)**			
**Direct effect**			
Childhood neglect→MC	0.11	<0.01	[0.034, 0.186]
**Indirect effect**			
Childhood neglect→	0.12	<0.001	[0.071, 0.150]
Machiavellianism→MC			
Childhood neglect→psychopathy→MC	0.03	<0.05	[0.004, 0.067]
Childhood neglect→narcissism→MC	0.04	<0.01	[0.019, 0.061]
Total indirect effect	0.18	<0.001	[0.123, 0.234]
**Males (*N* = 236)**			
**Direct effect**			
Childhood neglect→MC	0.14	<0.05	[0.023, 0.259]
**Indirect effect**			
Childhood neglect→	0.20	<0.001	[0.125, 0.287]
Machiavellianism→MC			
Childhood neglect→psychopathy→MC	0.01	=0.694	[-0.057, 0.084]
Childhood neglect→narcissism→MC	0.09	<0.001	[0.052, 0.155]
Total indirect effect	0.31	<0.001	[0.208, 0.418]

*MC, malevolent creativity; CI, confidence interval.*

For males, childhood neglect significantly positively predicted Machiavellianism (β = 0.39, *p* < 0.001), psychopathy (β = 0.45, *p* < 0.001), and narcissism (β = 0.32, *p* < 0.001). Machiavellianism (β = 0.50, *p* < 0.001) and narcissism (β = 0.29, *p* < 0.001) significantly positively predicted malevolent creativity. The direct effect of childhood neglect on malevolent creativity was significant (β = 0.14, *p* < 0.05). Therefore, the results of the indirect effects demonstrated that Machiavellianism (β = 0.20, *p* < 0.001) and narcissism (β = 0.09, *p* < 0.001) partially mediated the relationship between childhood neglect and malevolent creativity. Bootstrapping tests indicated that the mediating effects of Machiavellianism and narcissism among males were significant [95% CI = (0.125, 0.287) for Machiavellianism and 95% CI = (0.052, 0.155) for narcissism], but the mediating effect of psychopathy was not significant (see Figure 3 and Table 3).

Finally, we used multi-group SEM to test gender differences among direct and indirect pathways of the model. The Wald Test results showed that the indirect effect of childhood neglect on malevolent creativity through Machiavellianism (value = 13.43, df = 1, *p* < 0.001), psychopathy (value = 8.98, df = 1, *p* < 0.01), and narcissism (value = 9.56, df = 1, *p* < 0.01) changed significantly between male and female participants. Furthermore, the total indirect effect of the Dark Triad (value = 12.80, df = 1, *p* < 0.01) on the relationship between childhood neglect and malevolent creativity also changed significantly in male and female participants. However, the direct effect of childhood neglect (value = 1.36, df = 1, *p* = 0.24) on malevolent creativity did not change significantly between male and female.

## Discussion

While many studies have explored the predictors of malevolent creativity from environmental and individual aspects (Clark and James, 1999; Lee and Dow, 2011; Harris et al., 2013; Baas et al., 2019), the current study was the first to examine whether childhood neglect was associated with malevolent creativity in the general population and to examine the mediating effect of the Dark Triad traits on this relationship. Three important results were obtained from this study. First, the results confirmed that childhood neglect was positively related to malevolent creativity. Second, the Dark Triad personality traits mediated the relationship between childhood neglect and malevolent creativity. Third, childhood neglect had a stronger effect on malevolent creativity through the Dark Triad among males than females.

The results indicated that childhood neglect was positively associated with malevolent creativity. This means that individuals who experienced more neglect in childhood were more likely to engage in malevolent creativity behaviors in adulthood. The perspective was roughly consistent with previous research, which indicated that parental negligence encouraged antisocial behavior and reduced prosocial behavior among adolescents (Llorca et al., 2017). The present finding underlines that the relationship between the family environment and individual creativity development is complex. While beneficial family environments and growing experiences promote the development of benevolent creativity, harmful ones not simply damage its development but may facilitate the development of malevolent creativity. According to social information processing theory, individuals who have experienced more neglect in childhood may be more likely to perceive neutral social information as threatening information, which could induce hostile thought (Gawronski and Cesario, 2013) and readiness to fight (Mobbs et al., 2015). At the same time, the neglected individuals in the threatening information are more vulnerable, easily stressed, and depressed (Harkness et al., 2006; Infurna et al., 2016), and have difficulty in emotion recognition and regulation (Edwards et al., 2005; Young and Widom, 2015; Jennissen et al., 2016). More importantly, when immersed in negative emotions, individuals are more introspective, analytical, and persistent in their cognitive processes (De Dreu et al., 2012), which allows them to generate novel and useful ways to achieve their goals of hurting people or damaging society.

The fact that the Dark Triad Traits partially mediate the association between childhood neglect and malevolent creativity supports Hypothesis 2. In the present study, we found that Machiavellianism, psychopathy, and narcissism were positively related to malevolent creativity, which means that individuals with high levels of Dark Triad personality traits exhibit high levels of malevolent creativity. This finding is in line with Kapoor’s (2015) study, which suggests that the Dark Triad score predicts engagement in negative creativity. In addition, we found that childhood neglect was positively related to Dark Triad personality traits. The outcome means that individuals who experience more neglect in childhood are more likely to develop self-centered, callous, and manipulative personalities in adulthood. This result is consistent with previous research, which showed that poor parental care affects individuals’ personality development and creates a distrustful and malicious interpersonal style (Csathó and Birkás, 2018). Those who were neglected or received less attention as children predispose them to seek immediate rewards and develop ruthless and hostile personalities, reflecting a faster life strategy; a fast life strategy subsequently leads to more maladaptive behaviors such as exploitation and retaliation (McCullough et al., 2013), which also supports life history theory.

We should note from the results that Machiavellianism, psychopathy, and narcissism are separately mediating to varying degrees, which is mainly caused by their varying degrees of association with malevolent creativity. Possible explanations for this result are as follows. Machiavellianism is darker, more callous, and manipulative, but it has no consistent association with impulsivity and can even be associated with delayed gratification in the face of risk. Malevolent creativity requires deliberate, innovative, and secretive harm, so Machiavellianism may be closer to malevolent creativity. As dark and callous personality traits, psychopathy is associated with dysfunctional impulsivity, including poor self-control, various cognitive deficits, and more risk-taking, so psychopathy is closer to aggression (see Liu et al., 2019) than malevolent creativity. The “callousness” of narcissism is more about self-centeredness and is further correlated with functional impulsivity that involves venturesome social engagement, so narcissism may have a moderate correlation with malevolent creativity. This finding is consistent with studies of Jones and Paulhus (2011, 2017), which indicate that all three of the Dark Triad traits are associated with exploitative interpersonal behavior, but the motivations and tactics vary.

The results also show that the relationship among childhood neglect, Machiavellianism, narcissism, and malevolent creativity is stronger for male participants than for female participants, which provides partial support for Hypothesis 3. We interpret the result as follows: males with childhood neglect may develop higher levels of self-centeredness and manipulation and subsequently display higher levels of malevolent creativity than females. The outcome is consistent with previous research (Muris et al., 2017) that the Dark Triad traits are more common among men than women and partially confirms Lee and Dow’s (2011) finding that men are more malevolent than women. However, we were surprised to find that there is an association between psychopathy and malevolent creativity among female participants but not among males, which means that men with high levels of psychopathy may not exhibit malevolent creativity. Cale and Lilienfeld (2002) found in their study that males and females with psychopathy differ in the manifestation of specific antisocial behaviors. Their study suggests that psychopathic men are more likely to engage in unlawful behavior and have more traffic offenses than females, whereas psychopathic women are more likely to have relationship difficulties and exhibit lying than males. We, therefore, infer that psychopathic men show little malevolent creativity, possibly because they have less self-control and cognitive flexibility than women.

The results should be interpreted with caution because there were some limitations in the study. First, we conducted a cross-sectional study, so a causal relationship cannot be established. Longitudinal or experimental designs are needed to provide a step toward. Second, the participants recruited in the present study are undergraduates, who still belong to special groups compared with adults in society. Therefore, it is limited to explain and predict the malevolent creativity behavior of adults in society based on the results of this study. Future studies need to replicate the results in various groups of subjects. Three, there were lower alphas reliability of some subscales (e.g., psychopath) in the present study, which may result in biased estimates. Finally, we used self-reported measures for all variables, so participants may conceal or refuse to admit. Future studies could use multiple methods, such as parent reports to measure childhood neglect, teacher or peer reports to measure Dark Triad personality traits and experimental methods to measure malevolent creativity. Doing so improves the quality of the response data and provides more possible insights into the variables involved than those being studied in the current work.

Despite those caveats, the present study is the first to consider how childhood neglect is related to malevolent creativity by highlighting the roles of the Dark Triad personality traits. Since most prior studies focus on the influence of social atmosphere and general personality on malevolent creativity (Clark and James, 1999; Lee and Dow, 2011; Baas et al., 2019), this study extends previous studies and broadens people’s understanding of malevolent creativity to a certain degree. Our findings suggest that childhood experiences can be a predictor of malevolent creativity and that Dark Triad personality traits play an important role. Therefore, we should focus on groups that were neglected in childhood and those with a high level of Dark Triad personality traits and guide them to a proper understanding of neglect to mitigate their expression of malevolent creativity. Furthermore, interventions developed and implemented to reduce childhood neglect hold some promise of reducing malevolent creativity in adults, and adults who are aware of their malevolent creative tendencies should consider their childhood neglect and actively seek social support.

## Data Availability Statement

The raw data supporting the conclusions of this article will be made available by the authors, without undue reservation.

## Ethics Statement

The studies involving human participants were reviewed and approved by the Research Ethics Committee of Tianjin Normal University. The patients/participants provided their written informed consent to participate in this study.

## Author Contributions

XJ provided the idea, designed this study, and wrote the manuscript. QW wrote the manuscript and analyzed the data. LL contributed to data collection. All authors contributed to the article and approved the submitted version.

## Conflict of Interest

The authors declare that the research was conducted in the absence of any commercial or financial relationships that could be construed as a potential conflict of interest.
